# Feasibility of intensive prolonged exposure for PTSD: a pragmatic pilot study in Swedish public outpatient psychiatry

**DOI:** 10.1186/s12888-026-07836-1

**Published:** 2026-01-27

**Authors:** Sim Jamil, Christian Rück, Maria Bragesjö

**Affiliations:** Centre for Psychiatry Research, Department of Clinical Neuroscience, Karolinska Institutet, & Stockholm Health Care Services, Hälsovägen 13, Region Stockholm, Stockholm, 141 86 Sweden

**Keywords:** Posttraumatic stress disorder, Prolonged exposure, Intensive prolonged exposure, Intensive treatment, Trauma-focused cognitive behavior therapy

## Abstract

**Background:**

Prolonged exposure (PE) is an established treatment for PTSD, but outcomes are often limited by dropout and delivery constraints. This study evaluated the feasibility, acceptability, and preliminary effectiveness of intensive-format PE (I-PE) delivered as a pragmatic intervention in a Swedish psychiatric outpatient setting.

**Methods:**

Thirty-three adults meeting the diagnostic criteria for PTSD were enrolled in a single-center, uncontrolled pragmatic feasibility trial embedded in routine outpatient care in Sweden. I-PE comprised five consecutive days of therapist-guided treatment, followed by three individual booster sessions at weeks 2, 4, and 8. The primary objectives were to assess feasibility through reach, retention, treatment adherence, satisfaction, and safety. Clinical outcomes included clinician-rated PTSD severity with the Clinician-Administered PTSD Scale for DSM-5 at baseline and 6-month follow-up; self-reported PTSD with the PTSD Checklist for DSM-5 at baseline, posttreatment, and 6-month follow-up; complex PTSD symptoms with the International Trauma Questionnaire at baseline and posttreatment; and depression with the Patient Health Questionnaire-9 and quality of life with the EuroQol 5-Dimension at baseline, posttreatment, and 6-month follow-up. Analyses were conducted using mixed-effects linear models under an intention-to-treat framework.

**Results:**

I-PE proved both feasible and acceptable within routine care; 97% (32/33) completed the full protocol, and no serious adverse events were reported. Treatment satisfaction was high, with 90% of participants indicating that they would recommend I-PE to a friend in need of similar help. Substantial reductions in PTSD symptoms were observed on both clinician- and self-rated measures from baseline to the 6-month follow-up. The within-group effect sizes were large for clinician-rated symptoms (Cohen’s *d* = 2.05 at 6-month follow-up) and self-reported symptoms (Cohen’s *d* = 1.05 posttreatment; *d* = 1.24 at 6-month follow up).

**Conclusions:**

Pragmatically delivered, I-PE shows promise as a time-efficient alternative to weekly care, with low dropout rates, and warrants fully powered controlled trials to establish efficacy, durability, and cost-effectiveness.

**Trial registration:**

ClinicalTrials.gov (registration ID: NCT05207462) on December 22, 2021.

## Background

Posttraumatic stress disorder (PTSD) is common and impairing, often following a chronic course and incurring high functional and psychiatric burdens, underscoring the need for accessible, effective interventions. Prolonged exposure (PE) is a first-line treatment with strong empirical support [1] and is typically delivered in up to 15 weekly 90-minute sessions [2]. However, 22–29% of patients discontinue before treatment completion, reducing real-world effectiveness [3, 4]. Moreover, the weekly format places ongoing demands on patients balancing work, commuting, and caregiving responsibilities, as well as on clinics with limited capacity. It may also increase the risk of avoidance between sessions [5–7].

To address these challenges, modifications to the standard format have been proposed. Higher-frequency and compressed delivery formats have been suggested to improve feasibility and engagement in routine care. Although intensive formats may require temporary scheduling adjustments, they can reduce the overall treatment duration and minimize avoidance between sessions. A recent meta-analysis of randomized controlled trials found non-inferior efficacy for higher- versus standard-frequency psychological interventions, including in direct head-to-head comparisons of the same treatment delivered at different intensities [8–12]. Importantly, higher-frequency trauma-focused interventions were associated with lower all-cause dropout [11]. An earlier review of intensive programs reported large within-group effects and low dropout, although most studies were uncontrolled and follow-up periods inconsistent [13]. Furthermore, intensive trauma-focused programs integrating PE and EMDR have also demonstrated efficacy in individuals with complex PTSD [14]. Taken together, the literature suggests that intensive formats may yield comparable symptom reduction to standard delivery while improving acceptability. However, the definition of intensive formats varies substantially across studies, with differences in duration, session structure, and treatment components. Moreover, many trials have been conducted in veteran or residential settings or used multicomponent protocols, and evidence from routine outpatient psychiatry remains scarce.

This study aimed to develop and pragmatically deliver a PE-intensive format tailored for Swedish outpatient psychiatry, designed to run alongside ordinary clinic operations without overnight stays or adjunctive components. A single-center pragmatic feasibility trial was conducted with the primary objective of assessing feasibility and acceptability (reach, retention, adherence, satisfaction, safety), whereas secondary objectives concerned preliminary effects on PTSD, complex PTSD, depression, and quality of life.

## Methods

### Design

This single-center, uncontrolled feasibility trial was conducted at a publicly funded outpatient psychiatric clinic specializing in PTSD in Stockholm, Sweden. Recruitment took place between December 20, 2021, and January 16, 2023. The Swedish Ethical Review Authority approved the study (registration ID: 2021-06004-01). The study was registered with ClinicalTrials.gov (identifier: NCT05207462), with registration completed on December 22, 2021, prior to enrolling any participants. Informed consent was obtained from all participants.

### Participants

Eligibility criteria were set to ensure external validity by recruiting a representative sample of PTSD patients in outpatient psychiatric care in Sweden. Participants had to meet the full DSM-5 criteria for PTSD as assessed with the Clinician-Administered PTSD Scale for DSM-5 (CAPS-5; [15]), be 18 years or older, be proficient in Swedish, and, if taking psychotropic medication, have maintained a stable dose for ≥ 4 weeks prior to enrollment. Adjustments to medication during the trial were discouraged but permitted and did not result in exclusion; any changes were systematically recorded. The exclusion criteria included conditions requiring immediate clinical attention and judged likely to interfere with the ability to participate safely in PE (e.g., active manic episode, active psychotic episode, or acute suicide risk), concurrent trauma-focused psychological treatment, and circumstances involving ongoing threat or risk of continued harm (e.g., ongoing intimate partner violence).

### Recruitment

The study enrolled 33 participants from the waitlist of a specialized outpatient service for PTSD. Preliminarily eligible patients were contacted by the research team by telephone and, if interested, scheduled for a diagnostic visit with a psychologist. At that visit, participants received written information, and eligibility was assessed through a clinical interview, the CAPS-5 to confirm DSM-5 PTSD as the primary treatment focus [15], and a standard suicide risk assessment. Written informed consent was obtained before the baseline assessments and treatment started. Eligible participants completed self-reported baseline measures prior to beginning treatment.

### Therapists and assessors

Treatment was delivered by nine licensed clinical psychologists and four resident psychologists working at a specialized outpatient clinic for PTSD. In line with the pragmatic design, therapists represented the range of clinical experience typically found in routine care. All the therapists had prior experience providing weekly delivered prolonged exposure and had between 1 and 4 days of training in the protocol. They received an additional half-day study-specific protocol orientation. All the sessions were conducted on site at the recruiting clinic.

During the intensive week, therapists participated in daily group supervision provided by author MB to support treatment fidelity and clinical decision-making; thereafter, MB continued to provide weekly supervision throughout the booster phase. MB is a certified PE trainer with extensive clinical and supervisory experience. Therapist competence was therefore supported through prior PE training, study-specific orientation, and continuous supervision.

Follow-up assessments were conducted by study clinicians who were not involved in the participants’ treatment, together with master’s-level psychology students. Due to the pragmatic, uncontrolled pilot study design, assessors were not blinded to treatment allocation. All assessors received structured CAPS-5 training (1–2 days) and had access to supervision from author MB as needed. Interrater reliability was not formally assessed. All assessors received structured CAPS-5 training (1–2 days) and had access to supervision from author MB as needed to ensure consistency and accuracy across assessments.

### Treatment

The intensive format is an adaptation of the original PE protocol [2], which was codeveloped with clinicians at the recruiting site to align with routine outpatient care. The program consists of treatment delivered over five consecutive weekdays (Monday to Friday), each comprising 7–8 h of structured therapy, followed by three 60-minute booster sessions at weeks 2, 4, and 8. In total, the treatment period spanned nine weeks and incorporated three key modifications: (1) condensed delivery across one week with subsequent booster sessions; (2) integration of both individual and group-based components; and (3) all therapeutic activities were conducted onsite, with no homework assignments.

During the treatment week, the participants receive a total of nine individual 60-minute sessions and five 120-minute group sessions. Individual sessions focused on imaginal exposure and subsequent emotional and cognitive processing of traumatic memories. Group sessions were used for therapist-led psychoeducation, introduction of controlled breathing in the first session, discussion of the in vivo exposure rationale and planning, followed by individually tailored in vivo exposure exercises conducted primarily outside the group room. Each treatment day typically consists of two individual sessions (60 min each), one 120-minute group session, and two 60-minute group-based self-practice sessions during which participants listen primarily to recordings of their imaginal exposure but may also engage in in vivo practice.

From day two onward, group time is increasingly devoted to individually tailored in vivo exercises, which are typically conducted outside the clinic. These group sessions are led by multiple therapists to provide structure and support; however, personal trauma narratives are not shared in the group setting. The booster sessions focus on continued imaginal exposure, consolidation of treatment gains, relapse prevention, and planning for sustained self-guided exposure practice.

### Outcomes and measures

#### Primary outcomes: feasibility and acceptability

The primary outcomes were feasibility and acceptability. Consistent with Proctor et al. [16], feasibility was defined as the extent to which the intervention could be delivered as intended under routine conditions. This study focused on patient-level indicators: reach, retention, treatment adherence, satisfaction, and safety, with organizational and cost outcomes deferred to future implementation research.

Reach was defined as the proportion of applicants who completed eligibility assessment and enrolled.

Retention was operationalized as the proportion of enrolled participants completing the posttreatment and 6-month follow-up assessments.

Treatment adherence was defined as the proportion of participants classified as treatment completers, i.e., those who attended all five consecutive intensive treatment days and all three booster sessions (weeks 2, 4, and 8).

Acceptability, operationalized as perceived appropriateness and satisfaction with I-PE, was measured posttreatment with the Client Satisfaction Questionnaire (CSQ-8; [17]). Following Smith et al. [18], CSQ-8 scores were categorized as poor (8–13), fair (14–19), good (20–25), or excellent (26–32).

Safety was indexed by adverse events (AEs) and serious adverse events (SAEs) deemed related or possibly related to participation. AEs and SAEs were systematically monitored via the Safety Monitoring Uniform Report Form [19], which was completed daily during the intensive week, weekly during the booster phase, and once posttreatment. At each assessment, the participants indicated whether they had experienced any negative effects potentially attributable to study participation.

#### Secondary outcomes

PTSD symptom severity was clinician-rated at baseline and 6-month posttreatment with the Clinician-Administered PTSD Scale for DSM-5 (CAPS-5; [15]). The interview evaluates the 20 DSM-5 PTSD symptoms, each scored 0–4, yielding a total severity score of 0–80. In addition to a severity score, the CAPS-5 provides a diagnostic determination of PTSD based on symptom criteria over the past month [15]. Reliability analyses in a veteran sample indicate strong psychometric performance of the instrument: internal consistency was high (Cronbach’s α = 0.88), interrater reliability was excellent (ICC = 0.91), and test–retest reliability was acceptable (ICC = 0.78) [15].

Self-reported PTSD symptoms were assessed via the PTSD Checklist for DSM-5 (PCL-5; [20]), a 20-item measure aligned with the DSM-5 criteria. Items are rated on a 0–4 Likert scale, yielding a total score between 0 and 80, with higher scores indicating greater symptom severity. Psychometric evaluations of the PCL-5 indicate excellent internal consistency (α = 0.95) alongside robust test–retest reliability. A score of 29 or above has been suggested as a threshold for probable PTSD in Swedish samples [21]. Participants with a ≥ 10-point decrease from baseline on the PCL-5 were classified as responders.

Self-rated symptoms of PTSD and complex PTSD (CPTSD) according to the ICD-11 were assessed with the International Trauma Questionnaire (ITQ; Cloitre et al., 2018). The ITQ includes 12 items, six capturing core PTSD symptoms (re-experiencing, avoidance, sense of threat) and six assessing disturbances in self-organization (DSO; affect dysregulation, negative self-concept, and relational difficulties). Responses are scored between 0 and 4, with values ≥ 2 considered indicative of a symptom. The ITQ has shown good reliability and validity for assessing CPTSD according to the ICD-11 [22].

Depression symptoms were measured via the Patient Health Questionnaire-9 (PHQ-9; [23]), a 9-item self-report tool used to evaluate the frequency of DSM-IV depressive symptoms over the past two weeks. The items are rated from 0 to 3, resulting in a total score ranging from 0 to 27. The EuroQol five-dimensional questionnaire (EQ-5D; [24]) was used to evaluate health status across five domains: mobility, self-care, usual activities, pain/discomfort, and anxiety/depression. Each dimension is rated on a three-level scale, which is converted to an index score [24]. Self-reported data [20, 23, 24] were collected at baseline, posttreatment, and 6-month follow-up; the ITQ [22] was administered at baseline and posttreatment.

### Statistical analyses

All the statistical analyses were carried out in Python 3.9.6, applying an intention-to-treat approach, and the outcomes were analyzed with mixed-effects linear models implemented via the mixedlm function in the statsmodels.formula.api module (version 0.14.0). Time was specified as an independent variable, and secondary outcome measures (e.g., PTSD, depression, and quality of life) were treated as dependent variables. In all the models, time was entered as a fixed predictor, and subject-specific intercepts were modeled to account for correlations among repeated observations. To assess potential bias due to attrition on the CAPS-5 at the 6-month follow-up, a sensitivity analysis was conducted via multiple imputation by chained equations (MICE). Given the robustness of the findings, a single imputed dataset was used for analysis.

### Use of artificial intelligence

ChatGPT (GPT-4o; OpenAI) was used exclusively for language editing; no generative functions, including content creation, data analysis or interpretation were utilized.

## Results

### Sample characteristics

The participant characteristics are presented in Table 1. In summary, the sample was predominantly female, and the majority of participants had at least one comorbid psychiatric diagnosis. The participants reported diverse index traumas, most commonly sexual abuse. The baseline CAPS-5 scores reflected a clinically severe sample, with nearly half of the participants scoring 50 or above, a level typically associated with severe PTSD symptoms. On average, nearly two decades have passed since the index trauma.

**Table 1:** Demographic characteristics and clinical features of the participants (*N* = 33)

Gender, *n* (%)	Women Men	30 (91) 3 (9)
Age	Mean (SD) Range	35.78 (12.12) 18–62
Current psychiatric comorbidity, *n* (%)	Any Depression Anxiety disorder ADHD Personality disorder, any Autism spectrum disorder Sleep disorder Bipolar disorder, type 2 Eating disorder	21 (64%) 10 (30%) 8 (24%) 6 (18%) 4 (12%) 4 (12%) 3 (9%) 1 (3%) 1 (3%)
Type of index trauma, *n* (%)	Rape/sexual assault/abuse Physical assault Witnessed sudden death Explosion	20 (61%) 9 (27%) 3 (9%) 1 (3%)
Childhood index trauma < 18 years Adulthood index trauma > 18 years		23 (70%) 10 (30%)
Time from index trauma, mean year (SD), range	Mean (SD) Range	17.58 (13.80) 1–50
Baseline CAPS-5 ≥ 50	*n* (%)	16/33 (48%)
Baseline Self-rated Complex PTSD (ITQ)	*n* (%)	16/24 (67%)

Note. Participants could meet criteria for multiple diagnoses; totals may therefore exceed *N* = 33 and 100%

List of abbreviations: CAPS-5, Clinician-Administered PTSD Scale for DSM-5; ITQ, International Trauma Questionnaire

### Reach of the intervention

All applicants who expressed interest and completed the eligibility assessment were enrolled in the study (n = 33). The most frequently reported reasons for declining participation in the eligibility assessment were concerns about the intensity of the intervention (e.g., perceptions of it being ‘too demanding’ or ‘too difficult’), lack of interest in research participation, and scheduling constraints that prevented attendance during the treatment week.

### Retention and treatment adherence

At the 6-month follow-up, 21 of 33 participants (64%) completed the CAPS-5 interview.

For the self-reported PCL-5, 24 of 33 participants (73%) completed the posttreatment assessment, and 25 (76%) completed the 6-month follow-up. Adherence to the intensive regimen was excellent: 32 of 33 participants (97%) completed the full protocol, with one early discontinuation on Day 1 due to symptom exacerbation.

### Satisfaction

Treatment satisfaction, assessed with the CSQ-8, yielded a mean score of 24.86 (SD = 5.05; *n* = 29) out of 32. Overall, 83% rated the service quality as good or excellent, 79% felt the treatment aligned with the type of help they were seeking, and 90% would recommend it to a friend in need of similar help. In addition, 82% were mostly or very satisfied with the amount of help received, 86% expressed overall satisfaction, and 83% indicated that they would seek similar treatment again if needed.

### Safety

Seventeen of 33 participants (52%) reported minor adverse events, with a mean of 9.29 per participant (SD = 6.87). These were judged as treatment-related and possibly reflective of initial symptom exacerbation (e.g., emotional distress, fatigue, sleep disturbances, and headaches). Importantly, no serious adverse events occurred during the study.

### Secondary outcomes

Significant improvements were observed across both clinician- and self-reported PTSD measures, with large within-group effects sustained at six-month follow-up. At the 6-month follow-up, 8 of 21 participants (38%) met the diagnostic criteria for PTSD according to the CAPS-5. Sensitivity analyses using a linear mixed-effects model with imputed values indicated a reduction in CAPS-5 scores from a baseline mean of 46.73 to 24.19 at the 6-month follow-up (Cohen’s d = 1.8, *p* < .001). These findings were consistent with the primary analysis based on available (nonimputed) data and provide additional insight into the potential impact of missing data.

On the PCL-5, 80% of the participants met the responder criteria (≥ 10-point reduction). Complex PTSD symptoms (ITQ) also improved substantially, with only two participants meeting self-rated criteria at posttreatment. In addition to PTSD outcomes, depressive symptoms (PHQ-9) and quality of life (EQ-5D) also improved after treatment, with gains maintained at 6-month follow-up. See Table 2.

**Table 2:** Within-group effect sizes on clinical and self-report outcomes

Within-group effect sizes							
Outcome measure	Baseline	Post	6-month FU	Baseline to post	Within-group contrast baseline to post	Baseline to 6-month FU	Within-group contrast baseline to 6-month FU
Clinician-administered measure	M (SD), n	M (SD), n	M (SD), n	Cohen’s *d* (95% CI)	*P value* *P*	Cohen’s *d* (95% CI)	*P value*
CAPS-5	46.73 (11.29), *n* = 33		23.40 (14.50), *n* = 21			2.05 (1.61, 2.90)	< 0.001
Self-administered measures							
PCL-5	55.00 (11.79), *n* = 33	37.83 (17.28), *n* = 24	28.96 (15.90), *n* = 25	1.05 (0.51, 2.04)	< 0.001	1.24, (0.66, 2.62)	< 0.001
ITQ	35.25 (7.21) *n* = 24	19.29 (9.55), *n* = 14		1.45, (1.00, 2.40)	< 0.001		
PHQ-9	18.63 (6.16), *n* = 32,	12.23 (6.48), *n* = 22	12.35 (6.77), *n* = 23	0.78 (0.45, 1.22)	< 0.001	0.87 (0.47, 1.51)	< 0.001
EQ-5D	0.47 (0.21), *n* = 31	0.70 (0.17), *n* = 21	0.67 (0.21), *n* = 21	0.89, (0.50, 1.44)	< 0.001	1.21, (0.87, 1.81)	< 0.001

Note: Cohen’s d values represent within-group effect sizes based on the basis of paired comparisons. P- values were obtained from linear mixed-effects models, with time specified as a fixed effect and participant included as a random intercept. Abbreviations: FU = follow-up; CAPS-5 = Clinician-Administered PTSD Scale for DSM-5; PCL-5 = PTSD Checklist for DSM-5; ITQ = International Trauma Questionnaire; PHQ-9 = 9-item Patient Health Questionnaire; EQ-5D = EuroQol 5-Dimension

## Discussion

This pragmatic study investigated the feasibility, acceptability, and preliminary effectiveness of an intensive PE protocol for PTSD delivered under routine conditions in a Swedish outpatient psychiatric context. The results suggest that I-PE is both feasible for delivery within existing clinical workflows and well tolerated by patients with high treatment adherence, minimal dropout, and promising reductions in PTSD symptoms that were sustained at the 6-month follow-up. These findings contribute to the growing literature supporting intensive treatment models for PTSD and demonstrate their potential utility in public mental health services. Consistent with recent meta-analytic evidence [11], the results suggest that intensive formats may achieve comparable symptom reduction to standard weekly delivery while offering advantages in feasibility and patient acceptability.

### Feasibility and acceptability

I-PE proved feasible to implement within a publicly funded outpatient psychiatric clinic, even among patients characterized by high symptom severity. At baseline, nearly half of the participants (48%) scored 50 or above on the CAPS-5, and a substantial proportion met self-reported criteria for complex PTSD. All applicants who completed the eligibility assessment (*N* = 33) enrolled and initiated treatment, demonstrating both the feasibility of recruitment and the acceptability of the intensive format among patients seeking treatment.

Retention was moderate, with lower completion rates for clinician-administered interviews than for self-reported measures. Difficulties among clinicians in adhering to research procedures, such as inconsistent administration of measures despite clear guidance, contributed to data loss. This underscores the challenges of maintaining research rigor within routine care, even when treatment is delivered as intended. The lower retention rates for clinician-administered outcomes may also reflect outpatient barriers such as scheduling difficulties, reduced contact after the booster phase, or limited research infrastructure.

Adherence to treatment was high, with 97% (32/33) completing the full protocol, indicating a very low dropout rate, which is consistent with previous research on intensive treatment formats [11, 13]. This finding supports the notion that condensed delivery may reduce avoidance-related dropout and enhance engagement [11, 13].

The participants also reported high levels of satisfaction, with 90% indicating that they would recommend the intervention to others. These findings are consistent with meta-analytic evidence showing good acceptability and low dropout rates in intensive programs [11, 13] and suggest that such formats may be suitable for routine care where weekly therapy is difficult to deliver.

Over half of the participants reported minor adverse events (e.g., emotional distress, sleep disruption, and fatigue), but these events were transient and consistent with expected responses to exposure-based therapy, likely reflecting engagement with traumatic material rather than harm. Some patients may experience temporary symptom increases early in exposure therapy before improvement [2]. Importantly, no serious adverse events occurred, and only one participant discontinued after the first session because of symptom exacerbation. These findings support the acceptability of I-PE and align with previous research indicating that intensive trauma-focused interventions can be delivered safely [11, 13].

In summary, despite moderate retention, the study demonstrated favorable feasibility and acceptability outcomes in a group of outpatient psychiatric patients. These findings are consistent with earlier research on intensive trauma-focused interventions [11, 13] and extend the evidence base to routine outpatient psychiatric care.

### Changes in symptoms

Large and statistically significant improvements were observed in PTSD symptoms. CAPS-5 scores were nearly halved at the 6-month follow-up (Cohen’s d = 2.05), and PCL-5 scores showed comparably large improvements posttreatment and at follow-up. These effect sizes are consistent with previous trials of intensive PE formats and support the growing evidence that higher-frequency delivery can yield outcomes comparable to standard weekly PE while potentially improving treatment retention and feasibility [8, 10, 11].

Missing data posed challenges, with 36% attrition on clinician-administered outcomes at follow-up. However, sensitivity analyses using imputed data mirrored the primary findings, suggesting robust results.

Reductions were also observed in self-reported CPTSD symptoms (ITQ). At baseline, 16 participants met self-rated criteria for CPTSD, whereas only two met self-rated criteria for CPTSD posttreatment, suggesting that I-PE may be effective even for individuals with complex presentations. This pattern is consistent with previous findings demonstrating that intensive trauma-focused interventions can be effective for individuals with complex PTSD [14]. In addition, participants reported significant reductions in depression and improvements in quality of life, which were maintained at follow-up. Taken together, these results indicate that I-PE reduces core PTSD symptoms and supports recovery in CPTSD patients. Given the limited number of CPTSD studies, this is a valuable contribution.

Despite severe, complex presentations and long treatment delays, the intervention was well tolerated and produced substantial gains, challenging the view that intensive formats are ill suited to such patients. The observed improvements also indicate that I-PE may benefit individuals with disturbances in self-organization, a subgroup that remains underexplored in current research. While high comorbidity could influence engagement and response, it may likewise suggest that intensive formats are particularly valuable for patients with more severe clinical presentations.

Given the high prevalence of PTSD and limited resources in public health systems, time-efficient formats such as I-PE may offer a practical solution by reducing overall treatment duration and addressing barriers related to scheduling and caseloads. Future research should therefore prioritize replication in larger and more heterogeneous samples, as well as randomized controlled trials directly comparing I-PE with standard weekly PE and other active treatments. Such trials should extend follow-up periods and evaluate cost-effectiveness, scalability, and integration into broader health care systems. A randomized controlled trial directly comparing this I-PE format with weekly PE is currently underway [25].

### Strengths and limitations

This study has several notable strengths. It was embedded in routine outpatient psychiatric care, enhancing ecological validity and demonstrating that intensive PE can be delivered pragmatically within existing clinical services. Treatment adherence was exceptionally high, with nearly all participants completing the full protocol, and patient satisfaction was strong. The use of both clinician-rated and self-reported outcome measures, as well as sensitivity analyses with imputation, supports the robustness of the findings.

Several limitations should also be acknowledged. The absence of a control group restricts causal inference, as observed improvements could partly reflect natural recovery or nonspecific effects. The modest sample size and attrition on clinician-administered measures increase the uncertainty of effect estimates and limit generalizability; moreover, the study was not powered to detect small differences. Although supervision and protocol guidance were provided, therapist fidelity was not independently assessed. Follow-up assessments were conducted by study clinicians who were not involved in participants’ treatment but were also study therapists, which may have introduced potential allegiance bias, as assessors could consciously or unconsciously have a vested interest in favorable outcomes for the intervention. Future studies should employ fully independent assessors to minimize this risk.

The operationalization of reach as the proportion of applicants who completed eligibility assessment and enrolled follows conventions in feasibility research but may underestimate reach relative to routine clinical practice, where procedural and assessment demands are typically less extensive. Caution is therefore warranted when interpreting reach in relation to real-world implementation benchmarks.

Complex PTSD was assessed via self-report (ITQ) rather than structured ICD-11 interviews, which may have affected diagnostic precision. Future studies should incorporate structured interviews to more accurately assess CPTSD. Finally, the sample was predominantly female and recruited from a specialized outpatient clinic and characterized by high rates of childhood index trauma and comorbidity, which may reduce generalizability to more diverse populations and settings. Taken together, the results underscore the potential of I-PE as a feasible and acceptable intervention in routine care and highlight the need for rigorous trials to establish its long-term effectiveness and scalability. Consistent with recent meta-analytic evidence [11], the findings suggest that intensive formats demonstrate comparable efficacy to standard delivery while offering potential advantages in feasibility and patient acceptability.

## Conclusions

In summary, this pragmatic study indicates that I-PE is feasible and acceptable within Swedish outpatient psychiatry, with preliminary evidence of substantial and durable symptom improvements. High adherence, patient satisfaction, and sustained outcomes at 6-month follow-up underscore the potential of I-PE as a time-efficient alternative to standard weekly delivery. Supported by recent meta-analytic evidence [11] these findings suggest that intensive delivery may yield outcomes comparable to weekly PE while also improving retention.

Implemented pragmatically, I-PE appears compatible with outpatient services specialized in PTSD; however, the format with five consecutive treatment days plus follow-up sessions poses logistical demands, including temporary reallocation of clinical resources, dedicated therapist time for multiple daily sessions, and suitable space for parallel individual and group work. Future implementation studies should assess clinician workload, organizational readiness, and resource constraints to inform scalability and integration into routine care. Larger controlled trials are warranted to establish comparative efficacy, cost-effectiveness, and long-term outcomes.

## Data Availability

The datasets used and analysed during the current study are available from the corresponding author on reasonable request, in accordance with Swedish and EU data protection regulations.

## Abbreviations

AE: Adverse event
CAPS-5: Clinician-Administered PTSD Scale for DSM-5
CBT: Cognitive behavioral therapy
CPTSD: Complex posttraumatic stress disorder
CI: Confidence interval
CSQ-8: Client Satisfaction Questionnaire-8
DSM-5: Diagnostic and Statistical Manual of Mental Disorders, Fifth Edition
DSO: Disturbances in self-organization
EMDR: Eye Movement Desensitization and Reprocessing
EQ-5D: EuroQol 5-Dimension
FU: Follow-up
ICD-11: International Classification of Diseases, 11th Revision
ICC: Intraclass correlation coefficient
I-PE: Intensive Prolonged Exposure
ITQ: International Trauma Questionnaire
MICE: Multiple Imputation by Chained Equations
M: Mean
PE: Prolonged Exposure
PCL-5: PTSD Checklist for DSM-5
PHQ-9: Patient Health Questionnaire-9
PTSD: Posttraumatic stress disorder
SAE: Serious adverse event
SD: Standard deviation
P: p-value
